# Chromatic Pupillometry in Children

**DOI:** 10.3389/fneur.2018.00669

**Published:** 2018-08-17

**Authors:** Sylvain V. Crippa, Fatima Pedrosa Domellöf, Aki Kawasaki

**Affiliations:** ^1^Neuro-Ophthalmology Unit, Jules-Gonin Eye Hospital, University of Lausanne, Lausanne, Switzerland; ^2^Group for Retinal Disorder Research, Jules-Gonin Eye Hospital, University of Lausanne, Lausanne, Switzerland; ^3^Ophthalmology, Department of Clinical Science, Umeå University, Umeå, Sweden

**Keywords:** pupil, chromatic pupillometry, children, pupillary light reaction, growth

## Abstract

Chromatic pupillometry is a technique that is increasingly used to assess retinal disorders. As age may be one of the various factors which can influence the pupillary light reaction, this study aimed to evaluate the pupil responses to colored light stimuli in the pediatric population. Fifty-three children with normal vision and without any history of ocular disorders were tested with a portable pupillometer. Four test sequences were used: five dim blue (470 nm) stimuli presented in half log steps ranging from −3.15 to −1.15 log cd/m^2^ after 3 min of dark adaptation, five red (622 nm) stimuli of −1.15, −0.7, −0.15, 0.3, and 0.85 log cd/m^2^ after 1 min light adaptation, one bright blue stimulus of 2.2 log cd/m^2^ and one bright red of 2 log cd/m^2^. The results were grouped by age: a younger group included 27 children aged from 3 to 10 years old and an older group included 26 from 10 and 1 month to 18 years old. The younger group had a smaller pupil diameter after dark adaptation compared with the older group. A linear regression defining the photopic threshold showed that younger subjects had a higher threshold, e.g., needed a brighter red stimulus to evoke a threshold pupil response comparable that of subjects. Age thus seems to influence outer retinal sensitivity at least as evaluated by the pupillary photopic threshold intensity. The post-illumination pupillary reaction was used as a marker of intrinsic melanopsin activity and did not show any difference between the two age groups.

## Introduction

The use of colored light stimuli under conditions of dark and light adaptation facilitates rod versus cone mediation of retinal light signaling. More than 3 decades ago, Drs Lowenstein and Loewenfeld were recording the pupil response to focal green light flashes presented parafoveally at sub-photopic intensities to define the response curve of rods (1). In 1987, Birch and Birch (2) described a method using the steady-state pupil diameter after dark adaptation to determine the pupillometric threshold of rods, both in normal eyes and in eyes with retinal degeneration. The threshold was the retinal illumination necessary to evoke a criterion pupil response (defined as a decrease in pupil size by 1.0 mm). Adults with retinitis pigmentosa with reduced scotopic amplitude on electroretinography had elevated pupillometric rod threshold (mean 2.23 log units). Patients with nondetectable responses on electroretinography had pupil thresholds 3.27 log units higher than controls.

These early pioneering works which measured the pupil response to colored light stimuli under conditions of dark- and light-adaptation were the basis for quantifying rod and cone activity from the pupil light reflex. The discovery of the photopigment melanopsin and the identification of a non-rod, non-cone retinal photoreceptor have renewed interest in the pupil as a biomarker and have demonstrated that examination of the post-stimulus pupillary dynamics provides additional information about retinal light sensitivity.

In the 1960s, Bouma described that the steady-state pupil size that was largely determined by the scotopic spectral sensitivity using a large test field (3). Notably, he defined the pupil size against the intensity for different wavelengths and extracted the static pupillary sensitivity curve showing a peak at 490 nm. This is remarkably similar to the spectral sensitivity curve of melanopsin.

In terms of afferent pupillary signaling from the retina, melanopsin appears to be the predominant contribution for steady state pupil size and for sustained pupillary constriction following light stimulus offset. Various methods have been described to quantify this post-illumination pupil response (PIPR) which has a spectral sensitivity matching that of melanopsin (4–7).

Pupillometry using colored light stimulation has technologically advanced since the early experiments of the 60s and 70s. Chromatic pupillometry is now available as a small desktop or portable model (8–10). The simplicity of the technique broadens the patient population to be tested and is thus well-suited for use in patients with limited mobility. However, testing protocols still tend to be longer than most typical clinical tests and the dark adaptation needed to improve rod sensitivity could be difficult in patients with limited comprehension or attention span, such as patients with cognitive decline and young children. Yet it is the pediatric population in whom chromatic pupillometry may be a potentially important tool to evaluate outer and inner retinal activity in a variety of retinal and neurologic disorders (11).

Portable chromatic pupillometry for children may be an alternative to electroretinography for assessing photoreceptor function. The distinct advantage of pupillometry is that electrodes are not necessary. One foreseeable application is the school vision screening test. The ease of portable pupillometry permits on-site testing of children who fail the screening test. Chromatic pupillometry may also be used to monitor children with retinal degenerative disorders. In patients with endstage photoreceptor degeneration, chromatic pupillometry has been shown to be more sensitive than full-field electroretinography in detecting residual levels of cone function (12). Conversely chromatic pupillometry has promise as a tool to detect recovery of photoreceptor function in children who undergo gene therapy who are still too young to provide reliable responses to subjective tests of vision.

Thus, this pilot study was undertaken to evaluate chromatic pupillometry in children using a portable pupillometer.

## Subjects and method

The study was conducted according to the tenets of the Declaration of Helsinki and received authorization from the Regional Ethical Review Board for human research. Because study participants were under-age minors, one parent of each subject provided oral and written informed consent for participation in the study. Healthy children from families and acquaintances of the staff of the eye clinic of the University Hospital of Umeå (NUS) in Umeå were invited to participate in the study. Premature birth, a history of ocular trauma or diagnosis, use of ophthalmic or systemic medications were exclusionary criteria. Visual acuity, a Donders confrontation test, microscopic examination of anterior segment and examination of the macula and optic nerve head with 90D lens was performed and were normal for all children included in the study. No child was wearing glasses for refractive error at the time of the study. Refraction with cyclopentolate was not performed. Fifty-three children aged 3–18 years old were included. Because the axial length of the eye reaches its adult size at 10 years of age (13), the children were divided into two groups: those aged 10 years or less, here forth called the younger group and those over age 10 years called the older group. Detailed information was provided to the child and the accompanying parent prior to the recordings. The smaller children were given the opportunity to explore the environment and to “test” the different steps of the procedure before starting.

Pupil recordings were made using the IDMed Neurolight (Marseille, France) portable device. The light source is composed of three trichromatic light emitting diodes with a 6 log unit range of intensity placed in a kurbisfeld. For this study, only the blue (470 nm) and red (622 nm) lights were used. In this kurbisfeld, an infrared camera records the pupil continuously at 67 Hz with telecentric optics. A touchscreen graphical user interface is situated on the back of the stimulation chamber. It allows the examiner to enter the name of the subject and to select the pre-programmed stimulus sequence. This touchscreen offers a window where the pupil image is shown continuously during the recording. An occlusive rubber ocular is placed over the eye to be tested and the subject is instructed to look straight ahead. The examiner may stabilize head movement by placing a hand gently on the subject's forehead. In this study, the right eye was the tested eye.

We developed four test sequences (Supplemental Figure 1), modified from a previously-described stimulus protocol which was used to evaluate photoreceptor function in patients with rod-cone degeneration due to NR2E3 mutation (14). The relatively short times for dark and light adaptation in this study were selected from pre-study trial-and-error experiences noting child comfort and cooperation as well as the practicality of pupil testing in a clinical outpatient setting. We have retained the naming of the stimulus light sequence as “scotopic” and “photopic” for those following dark and light adaptation respectively. For this study, we use these light sequences with shortened adaptation times to grossly assess outer photoreceptor function: the scotopic sequence with blue lights biased toward rod function and the photopic sequence with red lights biased to cone function. We acknowledge that the shortened adaptation times used in this study are, however, not standard and not validated for true measuring of rod and cone function.

The first test sequence is performed after dark adaptation. For dark adaptation in this study, both eyes of the child are covered with sticky patches and the child sits quietly, accompanied by a parent if needed, in a dark, windowless room (0 cd/m2) for 3 min. The testing is performed in darkness as the pupilometer is placed over the right eye and the left (non-tested) eye remains under occlusion with a sticky patch. The pupil recording is started and the scotopic test sequence begins with 5 s of darkness (0 log cd/m2) followed by a series of five stimulations from a dim blue light having an intensity from −3.15 to −1.15 log cd/m^2^, in increasing half log steps. Each stimulation is 1 s in duration. The inter-stimulus interval is 3 s as this had been previously determined to be sufficient time to permit the pupil to return to baseline size before the arrival of the next light stimulus. The pupillometer is then removed from the right eye, the occlusive patch is removed from the left eye and the room light is turned on (900 lux) to start light adaptation for 1 min before the second test sequence. The second test sequence (photopic sequence) also begins with pupil recording during 5 s of darkness (0 log cd/m2) which is followed by a series of 5 stimulations from a red light having an intensity starting at −1.15 followed by −0.7, −0.15, 0.3, and 0.85 log cd/m^2^. Each stimulation is 1 s in duration and the inter-stimulus interval is 3 s. The eyes are again light adapted for 1 min before the third and before the fourth pupil tests sequences, each of which consists of a single light stimulation (1 s) having a high intensity and recording the pupillary response during the light stimulus and for 20 s in darkness after the light stimulus. The prolonged post-light recording of the pupil allows determination of the post-illumination pupil response (see PIPR calculation below). For the third test sequence, the stimulus is a blue light 160 cd/m^2^ and for the fourth test sequence, a red light stimulus 102 cd/m^2^, respectively 2.2 and 2 log cd/m2. In sum, the full protocol of this study consists of 12 stimuli divided into four test sequences; two with 5 stimuli and two with a single stimulus.

The pupil recordings were qualitatively assessed for validity of recording after each of the 4 testing sequences. For the first and second test sequences, pupil recordings having artifacts other than rapid blinks which occurred during 2 s of light stimulus onset were considered invalid responses and were removed from further analysis.

Scotopic and photopic test sequences having less than 4 valid responses out of five stimuli were considered invalid recordings. In cases where the first recording was deemed invalid, a second recording of the same test sequence was performed on the same eye (data not shown).

The pupil data were exported and analyzed in a spreadsheet (Microsoft Excel 2010; Microsoft, Redmond, WA). Blink artifacts were removed from the raw pupil tracings with a customized semi-automated filter function. The baseline pupil size (diameter) was determined from the first test sequence following dark-adaption and defined as the mean diameter during 250 ms just before the first light stimulus. This baseline pupil size was used to determine the pupil contraction amplitude. The maximal contraction amplitude is reported in this study as the maximal decrease in pupil size (in %) within 2 s of the light stimulus onset and calculated by the following formula: % maximal contraction amplitude at time x = ([baseline pupil diameter minus pupil diameter at time x]/[baseline pupil diameter]) × 100. A criterion level of 5% contraction amplitude was applied to distinguish evoked pupil responses from random noise.

The post illumination pupil response (PIPR) (7, 15) was calculated for the two last test sequences (single stimulus sequences having either a blue light or a red light). The PIPR (in %) was calculated as following: 100 – ([mean pupil diameter between 5.75 and 6.25 s after termination of the light stimulus/baseline pupil diameter] × 100) (6). The PIPR is a clinical marker of the melanopsin contribution to the pupil light response and is generally greater following a blue light stimulus compared to a red light stimulus as melanopsin has relatively poor sensitivity to long wavelength light (7).

Outcome parameters were compared using student *t*-test for normalized data. The significance threshold was defined as ≤0.05.

## Results

In the younger group (<10 years old) there were 9 boys and 18 girls and in the older group (>10 and <18 years old), 13 girls and 13 boys. The ages of children in the younger group ranged from 3 years and 4 months old to 10 years old (mean ± SD: 80 ± 23 months; the oldest of this group had her recording during her 10th anniversary month) and the older group were aged from 10 years and 1 month old to 17 years old and 10 months (153 ± 26 months).

The first test sequence was valid in every subject except two; one in the younger group and one in the older. For the second photopic test sequence, three children in the younger group had an invalid recording. For the two last test sequences, the older group had no difficulties, but four in the younger group had invalid recording due to prolonged eye closure: one with the bright blue stimulus of the third test sequence and three with both bright red and blue sequences.

All subjects had at least two valid sequences for analysis except one 9 year old subject who only had the 1st sequence deemed valid. Forty-five subjects could complete the full protocol (four test sequences); these were 21 of 27 (78%) in the younger group and 25 of 26 (96%) in the older group.

### Pupil size

The younger group showed a significantly smaller baseline pupil size, 7.2 ± 0.8 mm compared to the older group of 7.6 ± 0.8 mm (*p* = 0.03).

### Contraction amplitude

**Figure 1:**
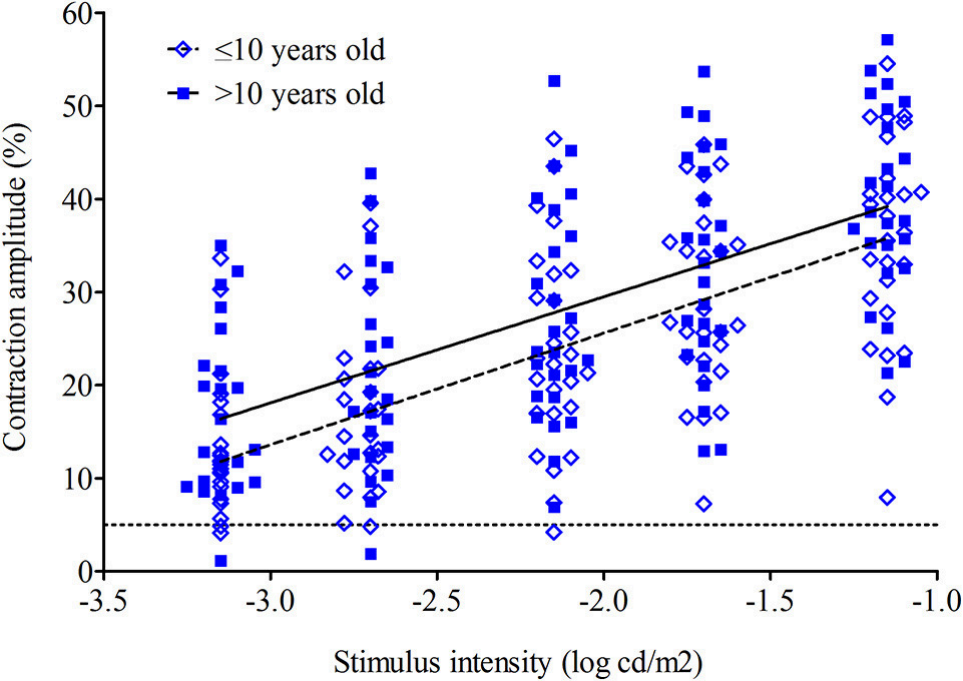
Maximal contraction amplitude to dim blue lights (scotopic test sequence) for 52 subjects (26 in younger group and 26 in older group). The mean contraction amplitude for younger (open diamonds) and older (closed squares) groups is plotted as a regression line across a range of intensities. The dashed line is the younger group; the solid line is the older group. The dotted line represents the threshold (5%) for defining an evoked pupil response.

In the scotopic test sequence, the pupil contraction amplitude increased with increasing stimulus intensity for both subject groups (Figure 1). This relationship appeared to be linear with a mean individual coefficient of determination of *r*^2^ = 0.92 ± 0.07 (0.92 ± 0.07 for the younger group and 0.93 ± 0.07 for the older group; *p* = 0.59). The slopes were compared and considered as equal (*p* = 0.61).

**Figure 2:**
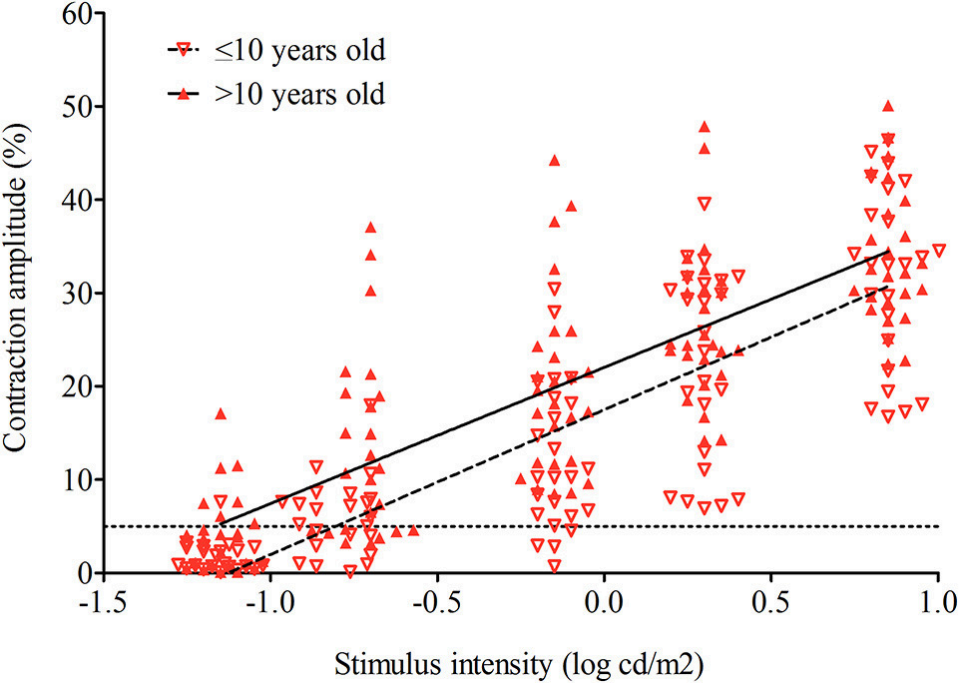
Maximal contraction amplitude to dim red lights (photopic test sequence) for 50 subjects (24 in younger group and 26 in older group). The mean contraction amplitude for younger (open triangles) and older (closed triangles) groups is plotted as a regression line across a range of intensities. The dashed line is the younger group; the solid line is the older group. The dotted line represents the threshold (5%) for defining an evoked pupil response.

For the photopic test sequence, the contraction amplitude increased with increasing stimulus intensity for both groups. This relationship appeared to be linear with a mean individual coefficient of determination of *r*^2^ = 0.88 ± 0.10 (0.86 ± 0.11 for the younger group and 0.90 ± 0.10 for the older group; *p* = 0.23); the slopes were compared and considered as equal (*p* = 0.91; Figure 2). Only one subject aged 6 years in the younger group had an observable pupil reaction (defined as >5% decrease in pupil diameter) to the dimmest red stimulus having intensity of −1.15 log cd/m^2^ whereas seven older subjects clearly show an evoked response at this dimmest red light stimulus. This and the generally lower pupil responses in the younger group suggest that threshold intensity for a pupil contraction to red light stimulation under conditions of light adaptation is slightly higher for younger children and overall retinal light sensitivity is lower.

### Threshold

From the regression lines determined for the scotopic and photopic test sequences, the threshold intensity could be extrapolated to the intercept at y = 5 which is the dimmest stimulus that produces a 5% pupil contraction. Only subjects with at least on 4 out of 5 valid stimuli were considered for this analysis. Twenty-six of 27 the younger group were included for the scotopic test sequence and 24 of 27 for the photopic test sequence. For the older group, these were 25 of 26 and 26 of 26 for the scotopic and photopic test sequences, respectively.

**Figure 3:**
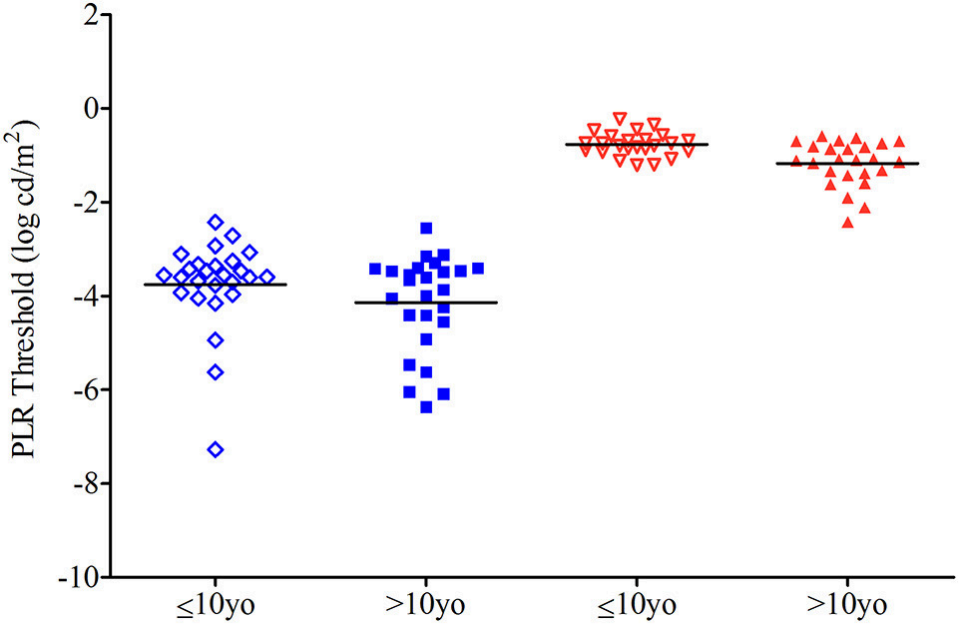
Dot plot of the threshold for 26 subjects of the younger group and 26 from the older one. Threshold intensity is the light intensity needed to produce a 5% contraction amplitude using a regression line. See Figures 1, 2. Horizontal bars represent the mean for younger (open symbols) and older (closed symbols) groups.

The mean threshold intensity for the scotopic test sequence is −3.75 ± 0.96 log cd/m^2^ for the younger group and −4.14 ± 1.03.log cd/m^2^ for the older group (*p* = 0.17) and for the photopic test sequence, the mean threshold is −0.77 ± 0.25 log cd/m^2^ for the younger group and −1.17 ± 0.48 log cd/m^2^ for the older group (*p* = <0.01; Figure 3).

### PIPR

**Figure 4:**
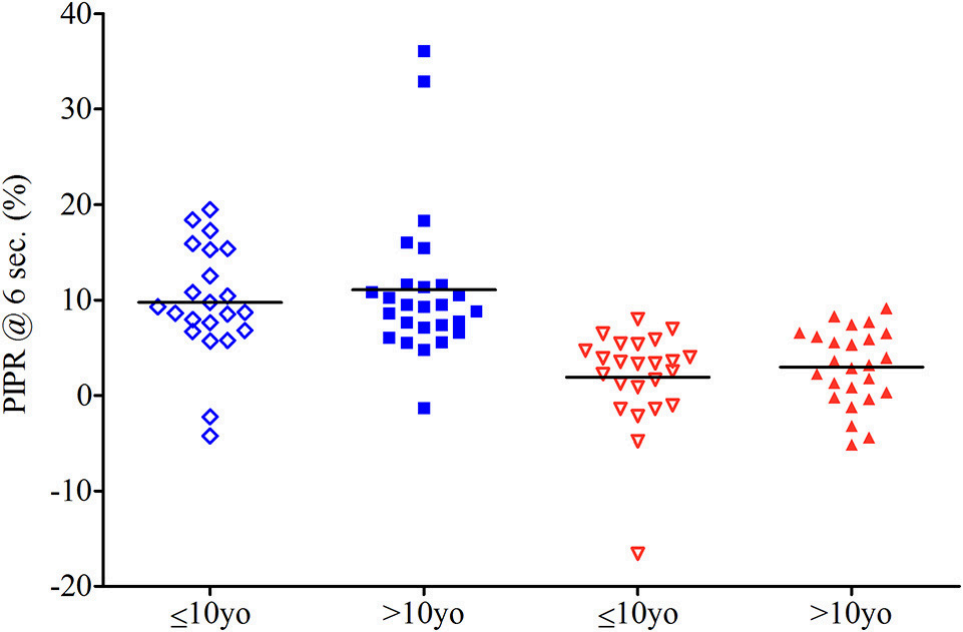
PIPR to blue light for 23 subjects in younger group and 26 subjects in older group and PIPR to red light for 24 in younger group and 26 in older group. Horizontal bars show the mean. Subjects in the younger group are shown in open diamond for the blue stimulation and open triangle for the red one. Those in the older group are respectively shown as closed squares and closed triangles.

The post-illumination pupil response for the third test sequence (single blue light stimulus) was larger (10.47 ± 7.04%), i.e., pupil size remained smaller after light offset, compared to that for the fourth test sequence having a single red light stimulus (2.45 ± 4.52%). This difference was statistically significant (*p* = <0.01). The older group showed a larger blue light PIPR in response (11.09 ± 7.92%) compared to younger group (9.75 ± 5.92%) but the difference was not statistically significant (*p* = 0.51). For the PIPR to bright red light, the older group showed a larger PIPR (2.97 ± 3.98%) compared to the younger group: 1.91 ± 5.05%; but again, the difference was not significant (*p* = 0.42; Figure 4).

The PIPR in response to the last red light test sequence for all children was expectedly very small (mean 2.45 ± 4.52%), and this is consistent with results from other studies made with primates and adult humans (6, 15, 16) The bright red light test sequence PIPR generally serves as a control stimulus in which melanopsin stimulation is presumed to be null-to-minimal. In order to relate the melanopsin contribution from the blue light stimulus to the control response, we also evaluated difference in between the blue light PIPR and the red light PIPR (difference PIPR). There is no difference in the difference PIPR between the younger groups (7.59 ± 8.81%) and the older group (8.21 ± 8.01%; *p* = 0.80).

## Discussion

This study used a portable, hand-held device to record the pupil response to a range of chromatic light stimuli in children. The ages of the children ranged from 3 years and 4 months to 17 years and 10 months old. The dark and light adaptation times were reduced in order to enhance subject cooperation. The two light-adapted test sequences using a single bright stimulus, one blue and one red, showed the most artifacts in the younger group. Three children for whom a valid recording was not possible in these two test sequence were aged of 6 years and 11 months, 8 years and 9 months, and 9 years and 8 months; showing that nonetheless the youngest of the participants (aged 3 years and 4 months) could cooperate enough to complete tests sequences with bright lights and a long post stimulus dark period.

The mean scotopic baseline pupil diameter was larger in the older group compared to the younger group; respectively a pupillary diameter of 7.2 ± 0.8 mm for the younger and 7.6 ± 0.8 mm for the older group (*p* = 0.03). This finding is consistent with the published literature (1, 17, 18).

From linear regression analysis, the intensity for a threshold pupil response (threshold intensity) was determined. We found the scotopic threshold showed a tendency to be higher in the younger group compared to the older group (−3.75 ± 0.96 log cd/m^2^ vs. −4.14 ± 1.03 log cd/m2, respectively, *p* = 0.17). The range of scotopic threshold values of the older group of children in this study were similar to those of adults (−4.7 ± 0.4 log cd/m2) who were tested with a ganzfeld stimulator and more rigorous protocol of light and dark adaptation (14).

The similarity in threshold intensity between children tested with the portable pupillometer and previously reported values in adults suggest that the abbreviated testing protocol of this study may be sufficient to assess rod-weighted pupil responses. For the threshold intensity of the photopic test sequence, we found a higher threshold in the younger group (−0.79 ± 0.28 log cd/m^2^) compared to the older group (−1.17 ± 0.48 log cd/m^2^) and this difference was significant (*p* = <0.01). Our finding that younger children have a higher scotopic and photopic threshold intensity may have importance when interpreting results of chromatic pupillometry for clinical purposes in children.

While it is beyond the scope of this study to examine the reason behind this age-related difference in threshold values, we may postulate on several possibilities for why the younger group requires a greater light intensity to evoke a minimum 5% pupillary contraction. These include: decreased neural signal from the retina to the olivary pretectal nucleus, greater supranuclear inhibition of the afferent pupillomotor signal at the level of the olivary pretectal nucleus, lesser neural signal from the Edinger-Westphal nucleus or greater mechanical resistance to pupillary movement at the level of the iris. We favor the first emitted hypothesis that decreased retinal light sensitivity related to ongoing postnatal retinal development is the basis for the higher thresholds seen in younger children. Anatomic and functional studies provide some support data (19–21).

From a histopathologic study, cone density at the fovea is 108,400/mm^2^ at age 3.8 years which is still far under the density of 208,200/mm^2^ in adults (19, 20). However, cone packing is completed before age 10 years after which the fovea attains its adult characteristics. From an electrophysiologic study, it has been shown that the rod and cone response (b-wave amplitude) increases between age 1 and 20 years with peak values occurring between age 10 and 20 years (21). Specifically, the rod b-wave median amplitude was 45% higher in children aged 10–20 years compared to those aged 1–10 years. Similarly the cone b-wave median amplitude and the cone a-wave median amplitude by electroretinography were larger in the older group by 21 and 26%, respectively. These anatomic and functional studies suggest that the outer retina in children under age 10 is still developing and is yet less light sensitive than a fully mature one.

In all four test sequences the older group had a larger pupillary contraction compared to the younger group. In the scotopic test sequence, the pupil contraction amplitude increased with increasing stimulus intensity for younger and older groups (Figure 1). This relationship appeared to be linear for both groups (younger group: slope 14.73 ± 5.19, *r*^2^ = 0.92 ± 0.07; older group: slope 14.87 ± 3.0, *r*^2^ = 0.93 ± 0.07). There was no difference in the slope or the variance between the two groups (*p* = 0.61 and *p* = 0.59, respectively).

For the photopic test sequence, the contraction amplitude increased with increasing stimulus intensity for younger and older groups (Figure 2). This relationship appeared to be linear for both groups (younger group: slope 12.11 ± 3.68, *r*^2^ = 0.86 ± 0.11; older group: slope 11.66 ± 2.35, *r*^2^ = 0.90 ± 0.10). There was no difference in the slope or the variance between the two groups, *p* = 0.91 and *p* = 0.23, respectively. Only one subject aged 6 years in the younger group had an observable pupil reaction (defined as >5% decrease in pupil diameter) to the dimmest red stimulus having intensity of −1.15 log cd/m^2^ whereas seven older subjects clearly show an evoked response at this dimmest red light stimulus. This and the generally lower pupil responses in the younger group suggest that threshold intensity for a pupil contraction to red light stimulation under conditions of light adaptation is slightly higher for younger children and overall retinal light sensitivity is lower.

The blue light PIPR in this study was relatively small, suggesting suboptimal stimulation of melanopsin. We selected the intensities for these two test sequences in part from a widely shared methodology (6) and in part from consideration for light tolerance in children. There were no differences between the two age groups in the PIPR determined from the single stimulus sequences. This is contrary to the age-related differences observed with the scotopic and photopic sequences. We can postulate that the absence of an age effect on PIPR indicates that melanopsin-mediated phototransduction matures early in human development. It is also possible that the PIPR determined by this methodology is not sensitive enough to detect developmental changes in melanopsin light sensitivity (22). For purposes of using PIPR as a clinical biomarker of melanopsin activity, we suggest that a larger population of children be tested so that trends in PIPR as a function of age can be verified as absent or present.

Overall, we found that portable pupillometry using four short test sequences permits, in children as young as age 3 years, a valid recording of pupil responses to light stimuli biased to favor one photoreceptive element: rods or cones or melanopsin. Ocular development, estimated by age, seems to influence outer retinal sensitivity at least as evaluated by the pupillary and in general, supports lower threshold intensity (greater light sensitivity of the outer retina) in children after age 10 years. The melanopsin sensitivity estimated from the blue light PIPR was not influenced by age.

## Data availability statement

The raw data supporting the conclusions of this manuscript will be made available by the authors, without undue reservation, to any qualified researcher.

## Author contributions

AK and FP conceived, designed, and supervised the project. FP performed the experiments. All authors participated in the analysis and interpretation of the experiments. SC and AK wrote the manuscript. All authors critically revised and approved the final manuscript version.

### Conflict of interest statement

The authors declare that the research was conducted in the absence of any commercial or financial relationships that could be construed as a potential conflict of interest.

## Supplementary material

Supplemental Figure 1 Graphical presentation of the full pupil protocol consisting of four test sequences. Each numbered line represents one of the four test sequences. The 1st test sequence starts after 3 min of dark adaptation (DA; 0 lux) whereas the following 3 sequences start after 1 min of light adaptation (LA; 900 lux). The pupil recording is represented by the x axis; the scale bar equals 1 s. Each pupil recording starts with 5 s of darkness before the first light stimulus is presented. The vertical bars represent the light stimuli; the intensity is given above each stimulus and each stimulus is 1 s in duration. The inter-stimulus interval for sequences 1 and 2 is 3 s. Recording during non-stimulus segments occurs in darkness (0 lux).
