# Supplementary figures and images for: Chromatic Pupillometry in Children
Source: Front Neurol. 2018 Aug 17;9:669. doi: 10.3389/fneur.2018.00669 (PMC6107754; doi:10.3389/fneur.2018.00669)

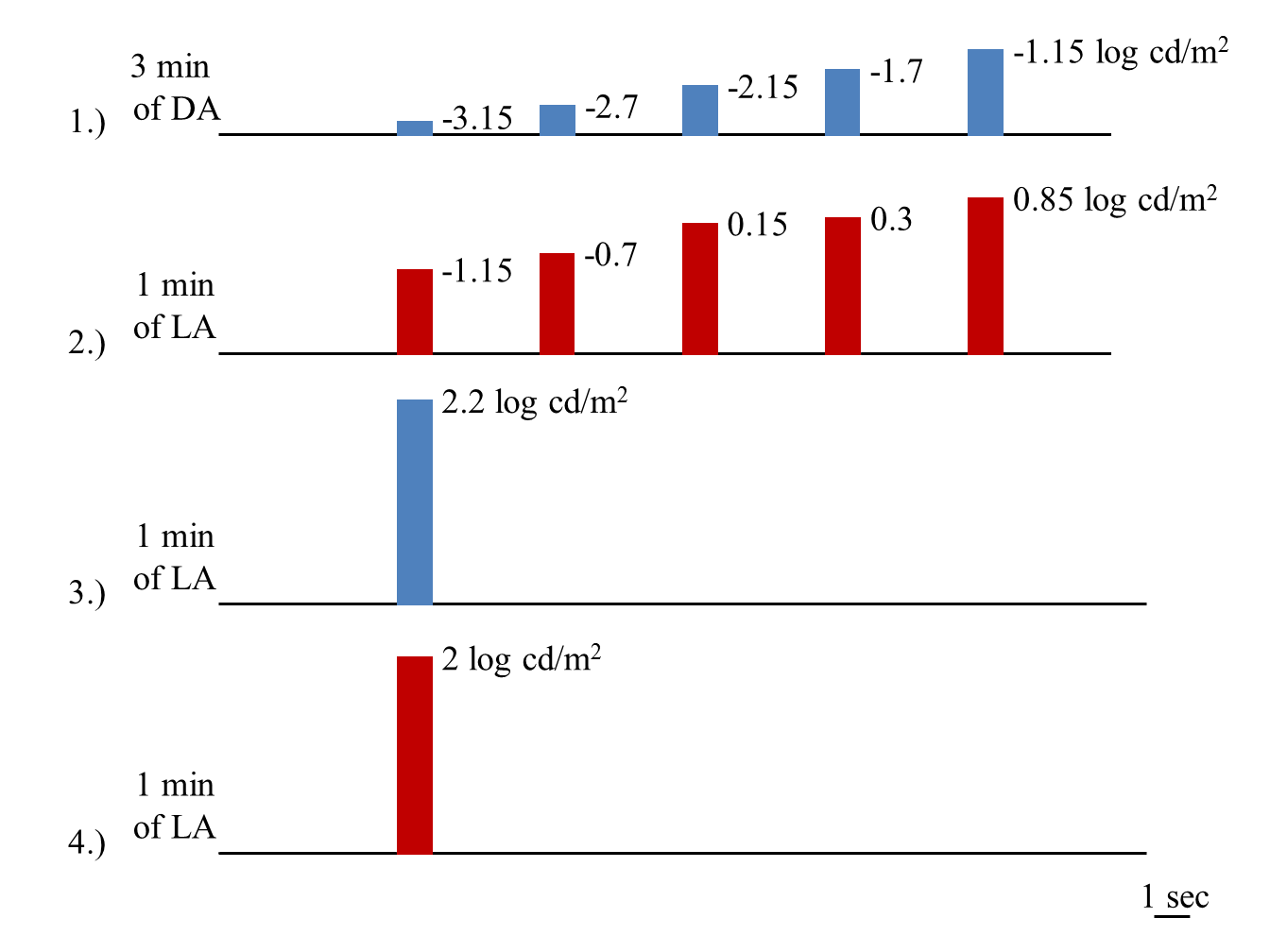

Supplement: Supplemental Figure 1 — Graphical presentation of the full pupil protocol consisting of four test sequences. Each numbered line represents one of the four test sequences. The 1st test sequence starts after 3 min of dark adaptation (DA; 0 lux) whereas the following 3 sequences start after 1 min of light adaptation (LA; 900 lux). The pupil recording is represented by the x axis; the scale bar equals 1 s. Each pupil recording starts with 5 s of darkness before the first light stimulus is presented. The vertical bars represent the light stimuli; the intensity is given above each stimulus and each stimulus is 1 s in duration. The inter-stimulus interval for sequences 1 and 2 is 3 s. Recording during non-stimulus segments occurs in darkness (0 lux). [file Image_1.TIF]
